# The genetic status of the Hungarian brown trout populations: exploration of a blind spot on the European map of *Salmo trutta* studies

**DOI:** 10.7717/peerj.5152

**Published:** 2018-09-21

**Authors:** Ágnes Ősz, Ákos Horváth, György Hoitsy, Dóra Kánainé Sipos, Szilvia Keszte, Anna Júlia Sáfrány, Saša Marić, Csaba Palkó, Balázs Tóth, Béla Urbányi, Balázs Kovács

**Affiliations:** 1 Department of Aquaculture, Institute of Aquaculture and Environmental Safety, Faculty of Agricultural and Environmental Sciences, Szent István University, Gödöllő, Hungary; 2 Hoitsy és Rieger Kft., Lillafüred, Hungary; 3 Institute of Zoology, Faculty of Biology, University of Belgrade, Belgrade, Serbia; 4 Department of Animal Nutrition, Institute of Animal Science, Faculty of Agricultural and Food Sciences, Széchenyi István University, Mosonmagyaróvár, Hungary; 5 Danube-Ipoly National Park Directorate, Budapest, Hungary

**Keywords:** Hybridization, Microsatellite, Salmo trutta, Lineage, Mitochondrial DNA, Sex ratio

## Abstract

**Background:**

Analyses of the control region sequences of European brown trout populations’ mitrochondrial DNA have revealed five main evolutionary lineages (Atlantic, Danubian, Mediterranean, Adriatic, Marble) mostly relating to the main water basins; however, the hybridization between lineages were increasingly reported. Due to the hydrogeography of Hungary, wild populations should theoretically belong to the Danubian lineage, however, this has not been verified by genetic studies.

**Methods:**

In our study multiple molecular marker sets (mitochondrial sequence, microsatellites, PCR-RFLP of nuclear markers and sex marker) were used to investigate the genetic composition and population genetics of the brown trout populations in two broodstocks, six wild streams in Hungary and one Serbian population.

**Results:**

The admixture of Atlantic and Danubian lineages in these populations, except the Serbian population with pure Danubian origin, was observed by control region sequences of mitochondrial DNA and PCR-RFLP markers in the nuclear genome, and one unpublished Danubian haplotype was found in Hungarian populations. A sex-specific marker revealed equal gender ratio in broodstocks and Kemence stream, whereas in other wild streams the proportion of female individuals were less than 50%. Structure and principal component analyses based on the alleles of microsatellite loci also revealed overlapping populations, however the populations were still significantly different from each other and were mostly in Hardy-Weinberg equilibrium.

**Discussion:**

Stocking and migration can have a significant genetic impact on trout populations of wild streams, however there are no guidelines or common practices for stocking of small streams in Hungary, thus the genetic background of these populations should be considered when developing conservation actions.

## Introduction

The family Salmonidae consists of three subfamilies (Coregoninae, Thymallinae and Salmoninae), which include a diverse group of fishes with sixty-eight species (Nelson, 2006). In the European continent, salmonids are native from Iceland to the Aral Sea and from Scandinavia to the Atlas Mountains of North Africa. Because of their economic importance, wide geographic distribution and phenotypic plasticity, salmonids are an extensively studied group regarding to their morphological (Norden, 1961; Stearley & Smith, 1993) and molecular variance (Apostolidis et al., 1997; Domanico, Phillips & Oakley, 1997; Kitano, Matsuoka & Saitou, 1997; Oohara, Sawano & Okazaki, 1997; Phillips & Oakley, 1997; Wang, Hard & Utter, 2002; Froufe et al., 2003; Crespi & Fulton, 2004; Aykanat et al., 2015). In spite of the abundance of information on salmonids, the phylogeographic map of their populations in incomplete.

Brown trout *(Salmo trutta)* is a native salmonid species in Eurasia (Behnke, 1986). It is also one of the most studied salmonids. In order to investigate the evolutionary history and phylogenetic relationships of this species, it was analysed by numerous molecular genetic markers and methods, including allozymes (Osinov, 1984; García-Marín & Pla, 1996), RFLPs (restriction fragment length polymorphism) (Morán, Pendás & García-Vázquez, 1996), PCR-RFLP (Marić et al., 2006a), microsatellite markers (Presa & Guyomard, 1998; Lerceteau-Köhler & Weiss, 2006), sequencing analyses of mitochondrial DNA (Bernatchez, Guyomard & Bonhomme, 1992; Giuffra, Bernatchez & Guyomard, 1994) as well as using of high-throughput techniques such as single nucleotide polymorphism analysis (Pustovrh, Snoj & Bajec, 2011; Sušnik Bajec et al., 2015) and next-generation sequencing (Sahoo et al., 2016). These studies have identified that the genetic background of European brown trout populations was related to various hydrogeographical areas. These genetic differences supposedly evolved during different colonization processes after the last glacial period (Hamilton et al., 1989; Weiss et al., 2000) and/or the geographical and reproductive isolation of the populations (Ryman, Allendorf & Ståhl, 1979).

Based on the sequence of mitochondrial DNA control region five main evolutionary lineages of brown trout were identified in Europe: Atlantic, Danubian, Mediterranean, Adriatic and Marble, mainly relating to the basins of main drainages. The Danubian lineage has colonised the rivers from the Black Sea to the Caspian and Aral basins, the Atlantic lineage has originally populated tributaries of the Atlantic basin, north Morocco and Sicily, while the distributions of the Mediterranean, Adriatic and Marble lineages overlap in the Mediterranean basin (Bernatchez, 2001).

Further analyses of European populations have revealed other subgroups of the brown trout: a different phylogenetic line in the Duero basin was suggested in the Iberian Peninsula (Suárez et al., 2001), different haplotypes and phenotypes were recognized in the Tigris Basin by the Persian Gulf (Tigris lineage) (Bardakci et al., 2006) and in Morocco (Dades lineage) (Snoj et al., 2011) and a Balkan cluster was identified in Southern Europe within the Mediterranean-Adriatic-Marble phylogenetic group (Marić et al., 2006b; Snoj et al., 2009). More detailed analyses of Lerceteau-Köhler et al. (2013) and Schenekar, Lerceteau-Köhler & Weiss (2014) revealed a natural process of ancestral hybridization between Atlantic and Danubian lineages in Austria (close to the natural boundary between the two drainages) caused by multiple colonization processes in the post-glacial period.

In addition to mitochondrial DNA, a nuclear genomic polymorphism of the lactate dehydrogenase gene was identified as a useful marker to distinguish the Atlantic alleles of brown trout from alleles of other origins (Ferguson & Fleming, 1983). The sequence of the somatolactin gene also contains several lineage-specific SNPs which are useful to assess the genetic composition of brown trout populations in case of this locus (Sušnik, Sivka & Snoj, 2008).

Currently, brown trout is cultured mainly for the stocking of natural streams managed by anglers, thus angling is a powerful driving force in the management of various populations. Many broodstocks used for the production of fish for stocking have originated from or mixed with the Atlantic lineage (Marić, Simonović & Razpet, 2010; Kohout et al., 2012), that led to the hybridization and introgression of the native and non-native populations as well as lineages in natural streams across Europe (Weiss et al., 2001; Snoj et al., 2002; Jug, Berrebi & Snoj, 2005; Almodóvar et al., 2006; Hansen & Mensberg, 2009). Based on these anthropogenic processes the protection of the local small populations has become increasingly important (Weiss et al., 2001; Weiss, 2005; Baric et al., 2010).

Although the origin and phylogeny of this species has been studied intensively in the countries around Hungary, the information available on Hungarian populations is sparse. The genetic background of farmed broodstocks are partly known and the results revealed that they are of mixed Atlantic and Danubian origin (Horváth et al., 2014). The history and formation of wild populations are also only partly known. Due to the hydrogreographical location of Hungary (all streams connected to the drainage of Danube River) it is expected that the local brown trout populations belong to the Danubian lineage. Because of the limited number of salmonid waters of Hungary, there are only a few natural populations and two hatcheries in the country. Fry and adult fish from the latter are stocked into natural streams. In addition, the few natural populations are located in the low mountain ranges, that are effectively isolated from each other by lowland areas where aquatic habitats are not suitable for salmonids. Thus, these populations are also characterized by a high degree of isolation from each other. The purpose of this study was to investigate the phylogenetic origin, the genetic background and structure of the Hungarian brown trout populations in natural watercourses (from here onwards referred to as wild populations) and farmed broodstocks. Various mitochondrial (control region) and nuclear genomic markers (LDH, SL, microsatellite loci) were selected according to studies that analyse populations in neighbouring countries. In order to reveal the sex ratio of these populations, a Y chromosome related gene was also analysed (Yano et al., 2013).

## Materials and Methods

### Sample collection and DNA extraction

**Figure 1:**
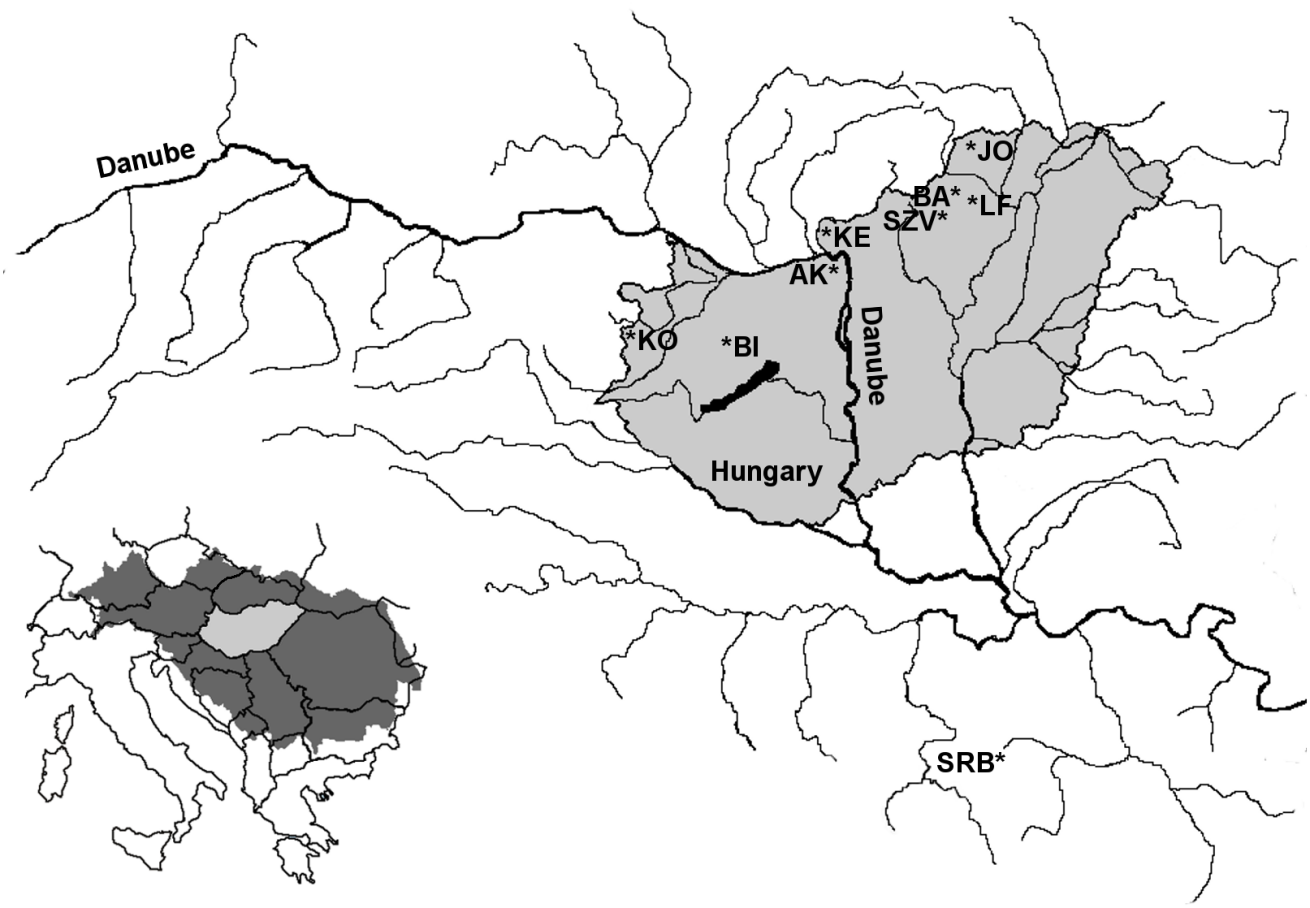
Location of the sampling area of farmed and wild brown trout populations within the Danubian water basin. LF-Lillafüred broodstock, SZV-Szilvásvárad broodstock, JO-Jósva stream, BA-Bán stream, KE-Kemence stream, AK-Apátkúti Stream, KO-Kölöntés stream, BI-Bittva stream, SRB-Serbian population in Panjica stream.

Altogether 888 brown trout were sampled from two brown trout broodstocks as well as from six wild streams (Bán, Jósva, Kemence, Apátkút, Kölöntés, Bittva) with poorly known stocking history and varying degree of angling pressure in the drainage system of the Danube in Hungary between 2011 and 2014 (Fig. 1, Table 1). Earlier all breerders (401 fishes) were sampled at the Lillafüred tout farm in order to create a marker-assisted breeding system in this broodstock (Horváth et al., 2014). Additional 243 breeder candidates were also sampled in the Lillafüred broodstock for this study; these fish originated from outside of the hatchery for refreshing the stock and were kept separately from the older breeders mentioned above. In addition, the entire broodstock from Szilvásvárad were also sampled in this study. As a control, a historically pure and geographically isolated, small Danubian population was sampled from the Panjica stream in Dobrače, Serbia in the Danubian drainage. The numbers of collected samples are presented in Table 1. In all sites, fish of various sizes were collected to avoid sampling of siblings and individuals from the same stocking event as much as possible. As the course of these streams is short, we attempted to collect samples from all sections. The fish were collected from the natural streams by the employees of the corresponding nature conservation agencies using electrofishing (Danube-Ipoly National Park Directorate, Őrségi National Park Directorate or Balaton-felvidéki National Park Directorate) or by the owners of the broodstocks using nets. On all locations, the fish were anesthetized in a 0.04% solution of 2-phenoxyethanol, then laid on a wet towel. A clip of the anal fin of each fish was taken and stored in 96% ethanol. Individuals of the two broodstocks were tagged with PIT (Passive Integrated Transponder) for later identification. After sample collection, all fishes were allowed to recover in anesthetic-free water and released back into their native streams or into the broodstock housing tanks. Genomic DNA was isolated from fin clips using the E.Z.N.A. Tissue DNA Kit (Omega Bio-tek, Norcross, GA, USA) according to the protocol of the manufacturer.

**Table 1:** Investigated farmed and wild populations of brown trout in Hungary; nine of these were newly analysed for this study and LF1 from Horváth et al. (2014).

**Abbreviations**	**Population**	**Status**	***N***	**N**_**seq**_	**Year of sampling**	**Coordinates of sampling locations**
LF1	Lillafüred 1.	Hatchery	401	41	2011	N48°07′03″ E20°34′07″
LF2	Lillafüred 2.	Hatchery	243	26	2013	N48°07′03″ E20°34′07″
SZV	Szilvásvárad	Hatchery	75	27	2014	N48°04′55″ E20°24′25″
BA	Bán	Wild	25	12	2012	N48°08′35″ E20°28′21″
JO	Jósva	Wild	33	16	2012	N48°28′56″ E20°32′49″
KE	Kemence	Wild	24	20	2012	N47°59′32″ E18°57′36″
AK	Apátkúti	Wild	50	28	2013	N47°44′53″ E18°59′40″
KO	Kölöntés	Wild	14	14	2013	N47°22′17″ E18°59′40″
BI	Bittva	Wild	9	9	2014	N47°13′19″ E17°33′21″
SRB	Panjica (Serbia)	Wild	14	12	2014	N43°39′34″ E20°04′20″

**Notes.**

*N* number of sampled and analysed individuals by microsatellites and PCR-RFLPs N_seq_ number of analysed samples of mitochondrial control region sequencing

### Analysis of mitochondrial and nuclear genome using DNA markers

The control region (CR) sequences of mitochondrial DNA (CR mtDNA), and PCR-RFLP of the same region (CR) as well as two nuclear genomic loci (lactate dehydrogenase: LDH; and somatolactin: SL) were used to distinguish the Atlantic and Danubian alleles/haplotypes. In addition, five microsatellite loci were used to analyse the genetic composition of the populations. Amplification and digestion methods were used according to Horváth et al. (2014). Sex of individuals was checked using a male determining gene (sdY) specific marker according to the protocol of Yano et al. (2013). Table 2 contains the applied marker sets, and amplification temperature of each loci. Briefly, for amplifying the DNA markers, mastermixes contained in 25 µl 1×PCR buffer with (NH_4_)_2_SO_4_ (Fermentas; Thermo Fisher Scientific, Waltham, MA, USA), 200 µM dNTP mix, 250 nM for each primers, 1,5 mM MgCl_2_, 100 ng template and 1 U Taq polymerase (Fermentas). The following PCR cycles were used: 3 min preamplification denaturation at 94 °C, then 30–60 s at 94 °C, 30–90 s at annealing temperature (30 s for sdY, 40 s for SsoSL438; 60 s for CRmtDNA, SL, LDH-C1, OMM1064 and BFRO002; 90 s for Ssa408uos and SsoSL417) and 1 min at 72 °C for 35 cycles. As a final step, products were fully elongated for 5 min at 72 °C. The reactions were checked on 1% of agarose gels, containing 0.5 µg/ml ethidium bromide. The microsatellite fragments were analysed on the ABI 3130 sequencer with POP7 polymer based on the various dyes.

**Table 2:** Applied genetic markers to analyses of brown trout populations in Hungarian hatcheries and wild streams as well as in one Serbian population.

**Marker**	**Type**	**Primers**	**Annealing temperature**	**RE/Dye**	**References**
CR mtDNA	Mitochondrial PCR-RFLP and sequence	F: 28RIBa: 5′-CACCCTTAACTCCCAAAGCTAAG-3′ R: HN20: 5′-GTGTTATGCTTTAGTTAAGC-3′	45 °C	Fnu4HI	HN20: Bernatchez & Danzmann, 1993; 28RIBa: Sušnik, Snoj & Dovč, 2001
LDH	Nuclear PCR-RFLP	F: 5′- GGCAGCCTCTTCCTCAAAACGCCCAA-3′ R: 5′- CAACCTGCTCTCTCCCTCCTGCTGACGAA-3′	58 °C	BslI	McMeel, Hoey & Ferguson, 2001
SL	Nuclear PCR-RFLP	F: 5′- TGGCCCGTTGAATCCATATAAAG-3′ R: 5′- ACTGTGAAACACTAAGCTCTCCA-3′	50 °C	MspI	Ford, 1998
BFRO002	Microsatellite	F: 5′-ATGTTTTTGACTGCACTATGTATTG-3′ R: 5′-GGAGATAAGAGTCAACGAGGC-3′	57 °C	NED	Sušnik et al., 1997
OMM1064	Microsatellite	F: 5′-AGAATGCTACTGGTGGCTGTATTGTGA-3′ R: 5′-TCTGAAAGACAGGTGGATGGTTCC-3′	57 °C	VIC	Rexroad et al., 2002
Ssa408uos	Microsatellite	F: 5′-AATGGATTACGGGTACGTTAGACA-3′ R: 5′-CTCTTGTGCAGGTTCTTCATCTGT-3′	57 °C	PET	Carney, Taggart & Høyheim, 2000
SsoSL417	Microsatellite	F: 5′-TTGTTCAGTGTATATGTGTCCCAT-3′ R: 5′-GATCTTCACTGCCACCTTATGACC-3′	57 °C	FAM	Slettan, Olsaker & Lie, 1995
SsoSL438	Microsatellite	F: 5′-GACAACACACAACCAAGGCAC-3′ R: 5′-TTATGCTAGGTCTTTATGCATTGT-3′	55 °C	FAM	Slettan, Olsaker & Lie, 1996
sdY	Sex related on Y chromosome	F: 5′-ATGGCTGACAGAGAGGCCAGAATCCAA-3′ R: 5′-CTTAAAACCACTCCACCCTCCAT-3′	60 °C	–	Yano et al., 2013

**Notes.**

RE restriction enzyme

#### PCR-RFLPs

Following the amplification, CRmtDNA fragments were digested with Fnu4HI (SatI) (Thermo Fisher Scientific; Waltham, MA, USA), LDH-C1 fragments with BslI (New England Biolabs, Ipswich, MA, USA) and the SL fragments with MspI (Thermo Fisher Scientific) restriction endonucleases. Endonuclease Fnu4HI generates a single cut at the polymorphic site C_434_ on the amplified CR mtDNA fragment of Atlantic haplotypes and no cut represents in Danubian haplotypes. Endonuclease BslI also generates a single cut on Atlantic lineage-specific allele of amplified LDH fragment at the polymorphic site G_353_ and no cut represents in Danubian alleles (McMeel, Hoey & Ferguson, 2001; Marić, Simonović & Razpet, 2010; Kohout et al., 2012). Based on the nucleotide polymorphism of the somatolactin gene between Atlantic (EU672412) and Danubian (EU672413) sequences in GenBank, endonuclease MspI generates no cut on Atlantic and a single cut at 212 bp on the Danubian lineage-specific allele of the SL sequences in GenBank. The alleles were separated on 2% agarose gels, containing 0.5 µg/ml ethidium bromide.

#### Sequencing analysis of the control region of mitochondrial DNA

For the comparison of populations based on haplotypes of CR mtDNA, randomly chosen samples (at least 10%) of each sample group were analysed by sequencing of the same control region used in RFLP (Table 1). For bi-directional sequencing, the same primer pair and protocol were applied as for CR mtDNA PCR-RFLP (Table 2). BigDye Terminator v3.1 Cycle Sequencing Kit (Applied Biosystems, Foster City, CA, USA) was used to determine the amplified CR mtDNA fragment sequences (according to the manufacturer’s recommendations). After the sequencing reaction, the product was ethanol-precipitated, dissolved in HiDi formamide (Applied Biosystems) and analysed on the ABI 3130 sequencer with POP7 polymer. All the rare haplotypes were re-sequenced twice to verify the results.

### Data analyses

The sequences of CR mtDNA were visualized, aligned and analysed by the MEGA 5 (Tamura et al., 2011) software using Danubian (AY185568) and Atlantic (AY185577) reference sequences (Duftner et al., 2003). Evolutionary divergence between the haplotypes was estimated with Tamura-3 parameter model (Tamura, 1992) chosen as the best-fit model method in same software. The haplotype (Hd) and nucleotide diversity (*π*) of the control region per populations were analysed in DnaSP 5.10.01 (Librado & Rozas, 2009). A haplotype network was built using the median joining algorithm in the program NETWORK 4.1.1.2 (Bandelt, Forster & Röhl, 1999).

In case of nuclear markers, the mean number of alleles (Nma), effective allele number (Neff), observed (Ho) and expected (He) heterozygosity per locus and per population were calculated using GeneAlEX 6.5 (Peakall & Smouse, 2012). In case of the microsatellite loci, the same software was used to reveal the private alleles of each populations. Allelic richness (Ar), FIS values and tests for standard deviations from Hardy-Weinberg expectations (HWE), pairwise Fst values between populations were calculated in FSTAT 2.9.3.2 (Goudet, 2001; Goudet, 1995). The significance levels for multiple comparisons were estimated using the sequential Bonferroni correction (Rice, 1989). For Neighbor-Joining (NJ) tree, STRUCTURE and PCA calculation only data of microsatellite loci were used. The Neighbor-Joining tree was prepared using POPULATIONS software (Langella, 2002) based on Da distance (Nei, Tajima & Tateno, 1983) using 2,000 bootstraps replicates. To explore the possible internal substructures of the populations we have used the STRUCTURE (Pritchard, Stephens & Donnelly, 2000) software with a Bayesian, Markov Chain Monte-Carlo (MCMC) approach set to 10,000–100,000 iterations for each probable genetic cluster (K: 1–12) with five replicates. The most probable number of genetic cluster (K) was determined based on the method of (Evanno, Regnaut & Goudet, 2005) using STRUCTURE Harvester (Earl & vonHoldt, 2012). For the visual analyses of the genetic variation among population principal component analysis (PCA) was made using R environment adegnet 2.0.1. package (Jombart et al., 2008). GeneAlEX 6 software was used for hierarchical analysis of molecular variance (AMOVA) to calculate the levels of genetic diversity among populations. The presence of null alleles of microsatellites was analysed using the software MICRO-CHECKER (Van Oosterhout et al., 2004) with 99% of confidence interval using 1,000 bootstraping replicates.

## Results

### Variance of mitochondrial DNA

#### PCR-RFLP analysis of mitochondrial control region

Digested fragment-length analysis of CR mtDNA revealed the pure Danubian origin of the Serbian control population, whereas the Atlantic lineage were found in all Hungarian sites (Table 3). There was one population where Danubian lineage dominated (KO stream, 64% of Danubian CR mtDNA), however, we found one population (BI stream) as well where only the Atlantic lineage was present in case of CR mtDNA. Based on the results of the sex-specific marker, the sex distributions were equal in the analysed broodstocks and KE stream, whereas in the other wild streams the proportions of female individuals were less than 50%. Additionally, the percentage of female individuals with Danubian lineage on mitochondrial DNA was also variable (0–53%) (Table 3).

**Table 3:** Proportion of the female individuals (Nf) and the Danubian lineages in cultured and wild brown trout populations in Hungary based on sdY and PCR-RFLP markers.

Population	*N*	Nf	CR mtDNA	*NfD*	LDH	SL
LF1	401	n.d.	0.25	***0.00***	37.00	22.10
LF2	243	49.00	49.17	***52.94***	**9.92**	33.88
SZV	75	51.00	22.67	*41.18*	21.33	22.67
BA	25	26.09	8.70	*50.00*	31.25	**34.09**
JO	33	36.36	9.09	*33.34*	10.61	17.19
KE	24	50.00	4.16	***0.00***	41.67	29.17
AK	50	44.00	34.00	*41.18*	19.00	18.00
KO	14	35.71	**64.29**	*33.34*	**42.86**	3.57
BI	9	22.22	**0.00**	***0.00***	22.22	**0.00**
SRB	14	42.86	100.00	*42.86*	100.00	100.00

**Notes.**

CR mtDNA mitochondrial control region LDH lactate-dehydrogenase SL somatolactin *N* sample size Nf Percentage of female individuals of population NfD Percentage of female individuals with Danubian mitochondrial lineage in each population n.d. no data. Minimum and maximum values are written in bold type

#### Sequence analysis of mitochondrial control region

The alignment of the 205 CR mtDNA sequences have provided 753 bp comparable sequences with 11 polymorphic sites. The sequences of nine haplotypes are accessible in Nucleotide database of National Center for Biotechnology Information (NCBI) (https://www.ncbi.nlm.nih.gov/) with accession numbers from MG751088 to MG751096. The analysis of sequence variation has revealed nine haplotypes (Table 4), six of which were assigned to the Danubian and three to the Atlantic lineage. There was no conflict between the results of mitochondrial PCR-RFLP and sequence analyses in any of the analysed individuals.

**Table 4:** The number of the identified CR mtDNA haplotypes in cultured and wild brown trout stocks in Hungary based on 753 bp of control region.

Population	*N*	Da1 MG751088	Da2 MG751093	Da3 MG751096	Da4 MG751094	Da5 MG751090	Da6 MG751095	At1 MG751089	At2 MG751091	At3 MG751092	Hd	*π*
LF1	*41*				1			36	4		0.224	0.001
LF2	*26*		21					5			0.323	0.003
SZV	*27*	8	4		1	3		11			0.738	0.006
BA	*12*	2						10			0.303	0.003
JO	*16*	3						1	9	3	0.650	0.004
KE	*20*						**1**	19			0.100	0.001
AK	*28*	15		2				11			0.574	0.003
KO	*14*	6	3					2	3		0.758	0.005
BI	*9*							9			0.000	0.000
SRB	*12*	12									0.000	0.000
Total	*205*	46	28	2	2	3	**1**	104	16	3	0.670	0.005

**Notes.**

The GenBank accession numbers are shown and the newly described haplotype is written in bold type.

*N* sample size Da Danubian haplotypes At Atlantic haplotypes Hd Haplotype diversity *π* nucleotide diversity

**Figure 2:**
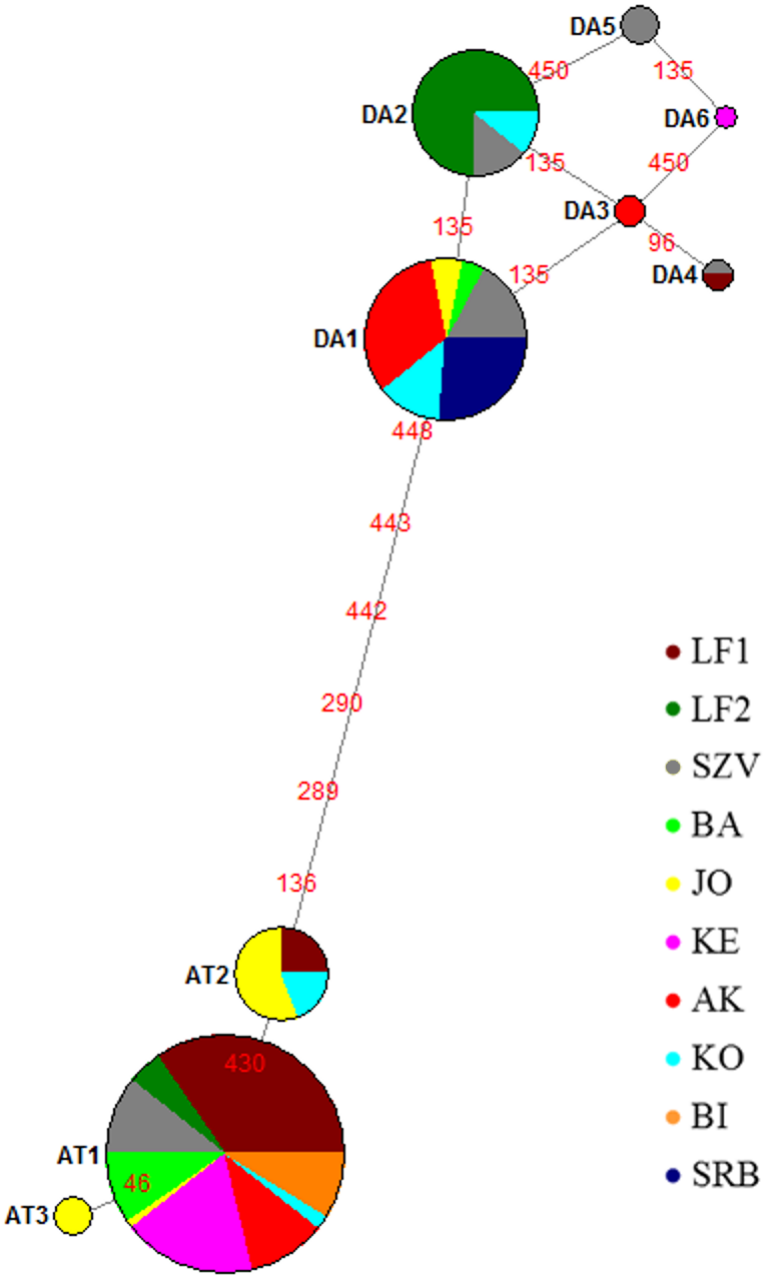
Median joining network of mtDNA haplotypes of brown trout samples collected from Hungarian wild streams (BA, JO, KE, AK, KO, BI), hatcheries (LF1-2 and SZV) and one wild population in Serbia (SRB). Red numbers refer to the mutation position in sequences between haplotypes; size of the circles corresponds to the haplotype frequencies in this sample set. Da, Danubian haplotypes; At, Atlantic haplotypes.

The median-joining network (Fig. 2) indicated two haplotype groups: Atlantic and Danubian. The Atlantic group consisted of all Atlantic haplotypes and the Danubian included all Danubian haplotypes. One of the Danubian haplotypes (Da6 GenBank accession no. MG751095) has not been described previously and was represented in only one individual of the KE stream. This haplotype also belongs to the Danubian group based on the median-joining network. The average divergence within the Danubian group was 0.0021, within the Atlantic group 0.0017 and between the groups 0.0112. The divergence of the newly found Da6 haplotype was 0.0013 from Da3, Da5 and 0.0027 from other Danubian haplotypes (Table S1). The median joining network revealed mixing of the lineages within the populations except the Serbian one as well as showed differences between the genetic composition of LF1 and LF2 broodstocks based on analysed sequences of these stocks.

The most abundant Danubian haplotype (Da1) appeared, beside the Serbian control, in the wild populations and the SZV broodstock. Da2 haplotype was detected in both hatcheries and one wild population while Da3 in one wild stream as a private haplotype with low frequency. Da4 haplotype was represented only in the hatcheries and Da5 haplotype was detected only in the SZV broodstock. The most frequent Atlantic haplotype (At1) was found in all populations with the exception of the Serbian control. In addition, two further Atlantic haplotypes were detected in the Hungarian populations, At2 haplotype was represented in the JO and KO streams as well as the LF broodstock 1, and the At3 Atlantic haplotype was found only in the JO stream as a private haplotype of this stock. The haplotype diversity of the sample set was 0.670 and the nucleotide diversity 0.005.

### Variance of nuclear DNA

#### Distribution of lineages in nuclear PCR-RFLP loci

Within nuclear markers, LDH and SL loci were analysed using digested fragment-length polymorphism. These loci also verified the pure Danubian origin of the Serbian control population, whereas varying proportions of both lineages were found in all Hungarian sites (Table 3). In both PCR-RFLP loci, the Atlantic lineage prevailed in each Hungarian population. There was one population (BI stream) where only the Atlantic lineage were presented in case of SL loci, but analysis of the LDH locus revealed 22% of Danubian alleles. Significant differences were found between the lineage frequencies of the two LF broodstocks.

#### Population structure and differentiation in nuclear loci

*F*_IS_ values of nuclear PCR-RFLP loci indicated a significant excess of heterozygosity in LF broodstock 1 and KE stream at *p* < 0.01 (Table 5), but this variance was caused by the strong deviation of SL locus (Table S2). Mean values of allelic richness (Ar) for each populations ranged from 1.000 to 2.000 and sample size-dependent effective allele number (Neff) values ranged from 1.000 to 1.825 (Table 5). Analysis of total *F*_IS_ values of microsatellite loci revealed no significant deviation in heterozygosity levels in any of the investigated populations, although the values are indicating weak heterozygosity degradation in three wild stocks: JO, KE and AK. Mean values of Ar for each population ranged from 4.805 to 7.276 and sample size dependent Neff values ranged from 2.118 to 7.446. Private alleles were found in all populations. Altogether 42 private alleles were observed with variable frequencies (0.001–0.362), which were mainly related to OMM1064 and Ssa408uos loci. The highest number (11) of private alleles was revealed in the two Lillafüred broodstocks (Table S3). Evidence of a null allele was found only at locus SsoSL417 in case of population LF1. In addition, this marker shows significant deviation from the Hardy-Weinberg Equilibrium (HWE) in LF broodstock 1.

**Table 5:** Summary of the population genetic analyses of two nuclear PCR-RFLP and five microsatellite (MS) loci for cultured and wild brown trout populations in Hungary.

Pop.	*N*	Loci	Nma	Neff	Ar		He	Ho	FIS	Sign.
**LF 1**	401	PCR-RFLPs	2	1.699	1.995		0.4057	0.4414	−0.0880	**
		MS loci	16.20	6.917	6.901		0.7721	0.7826	−0.0140	ns
**LF 2**	243	PCR-RFLPs	2	1.515	1.927		0.3140	0.3058	0.0260	ns
		MS loci	18.60	7.004	6.848		0.7400	0.7805	−0.0550	ns
**SZV**	75	PCR-RFLPs	2	1.523	1.992		0.3454	0.3733	−0.0810	ns
		MS loci	10.25	6.271	7.276		0.7621	0.7646	−0.0030	ns
**BA**	25	PCR-RFLPs	2	1.785	2.000		0.4493	0.5208	−0.1630	ns
		MS loci	10.50	7.145	7.102		0.8121	0.8835	−0.0910	ns
**JO**	33	PCR-RFLPs	2	1.316	1.944		0.2409	0.2779	−0.1570	ns
		MS loci	12.25	7.446	6.866		0.6911	0.6798	0.0160	ns
**KE**	24	PCR-RFLPs	2	1.825	2.000		0.4592	0.7083	−0.5610	***
		MS loci	6.75	3.764	4.815		0.6852	0.6917	−0.0100	ns
**AK**	50	PCR-RFLPs	2	1.432	1.983		0.3045	0.2900	0.0480	ns
		MS loci	8.250	4.841	5.613		0.6757	0.6708	0.0070	ns
**KO**	14	PCR-RFLPs	2	1.517	1.882		0.2897	0.3214	−0.1140	ns
		MS loci	5.750	3.993	5.772		0.6292	0.6846	−0.0930	ns
**BI**	9	PCR-RFLPs	1.5	1.264	1.500		0.1830	0.2222	−0.2310	ns
		MS loci	4.750	3.415	5.000		0.7320	0.7111	0.0300	ns
**SRB**	14	PCR-RFLPs	1	1.000	1.000		0.0000	0.0000	NA	NA
		MS loci	3.750	2.118	4.805		0.5651	0.5714	−0.0120	ns

**Notes.**

Pop. population *N* number of samples Nma mean number of alleles Neff effective allele number Ar Allelic richness He expected heterozigosity Ho observed heterozigosity FIS inbreeding coefficient Sign significant deviation of FIS NA not available ns not significant

** *p* < 0.01.

*** *p* < 0.001.

Pairwise *F*_ST_ values of microsatellite loci revealed low to intermediate levels of genetic variation among Hungarian populations (0.042–0.217) and intermediate levels between Hungarian populations and the Serbian control (0.222–0.369) (Table 6). The mean *F*_ST_ value for all populations was 0.110 (±0.039 SD). Beside the Serbian control, BI and KO streams were the most divergent sites among the Hungarian populations. All *F*_ST_ values of microsatellite analyses showed a significant divergence among populations. In case of the nuclear PCR-RFLP markers only the Serbian control was significantly divergent from all other populations and was highly distinct from them (0.558–0.920). Within the Hungarian populations low and intermediate levels of significant genetic divergence were found (0.027–0.231) and again, the BI, KO and KE streams were the most divergent sites.

**Table 6:** Pairwise *F*_ST_ divergences between the analysed brown trout populations based on microsatellite (below diagonal) and nuclear PCR-RFLP data (above diagonal) and their significance level.

	LF1	LF2	SZV	BA	JO	KE	AK	KO	BI	SRB
LF1		0.104^***^	0.027^*^	0.011	0.078^**^	0.043	0.037^*^	0.029	0.059	0.558^***^
LF2	0.075^***^		0.034^**^	0.055	0.036	0.052	0.045^*^	0.231^***^	0.153^*^	0.675^***^
SZV	0.060^***^	0.047^***^		0.018	0.013	0.044	0.004	0.094	0.048	0.674^***^
BA	0.085^***^	0.086^***^	0.063^***^		0.086^*^	0.007	0.042	0.102	0.119	0.615^***^
JO	0.076^***^	0.106^***^	0.042^***^	0.082^***^		0.116^**^	**0.001**	0.180^*^	0.061	0.814^***^
KE	0.132^***^	0.099^***^	0.100^***^	0.081^***^	0.154^***^		0.076	0.162^***^	0.178^***^	0.592^***^
AK	0.130^***^	0.075^***^	0.067^***^	0.089^***^	0.105^***^	0.136^***^		0.096	0.024	0.733^***^
KO	0.162^***^	0.156^***^	0.125^***^	0.119^***^	0.126^***^	0.156^***^	0.131^***^		0.047	0.812^***^
BI	0.108^***^	0.125^***^	0.098^***^	0.096^***^	0.125^***^	0.157^***^	0.184^***^	0.217^***^		0.920^***^
SRB	0.222^***^	0.247^***^	0.235^***^	0.234^***^	0.321^***^	0.287^***^	0.293^***^	0.369^***^	0.255^***^	

**Notes.**

*F*_ST_: **F**ixation index for **S**ubpopulation within the **T**otal population. Hungarian wild populations: BA, JO, KE, AK, KO, BI; Serbian wild popuation: SRB; broodstocks: LF1-2 and SZV. Minimum and maximum values within the Hungarian sites are written in bold type; maximum value within the whole sample set is bold and underlined.

* *p* < 0.05.

** *p* < 0.01.

*** *p* < 0.001.

**Figure 3:**
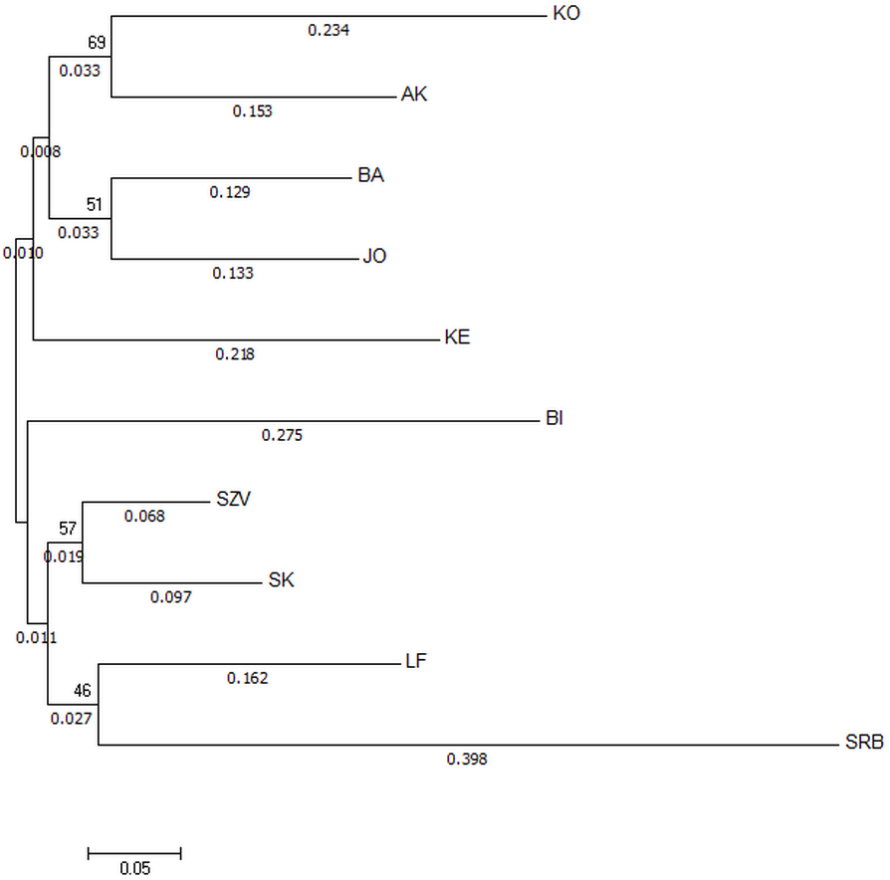
Neighbor-joining tree based on Da distances of microsatellite allele frequency of brown trout samples collected from Hungarian wild streams and hatcheries (LF, SZV) and a wild Serbian one (SRB). The numbers above the nodes indicate percent bootstrap support for each node over 2,000 replications. Only values over 50% are shown. The numbers under the nodes show the distance from node.

The Nei’s Da genetic distance based NJ tree of populations (Fig. 3) also supported that the most genetically distinct group was the Serbian control. The tree showed distance between the wild Hungarian populations and the hatcheries. The populations in the BI and KO streams were the most divergent ones among the Hungarian wild sites.

**Figure 4:**
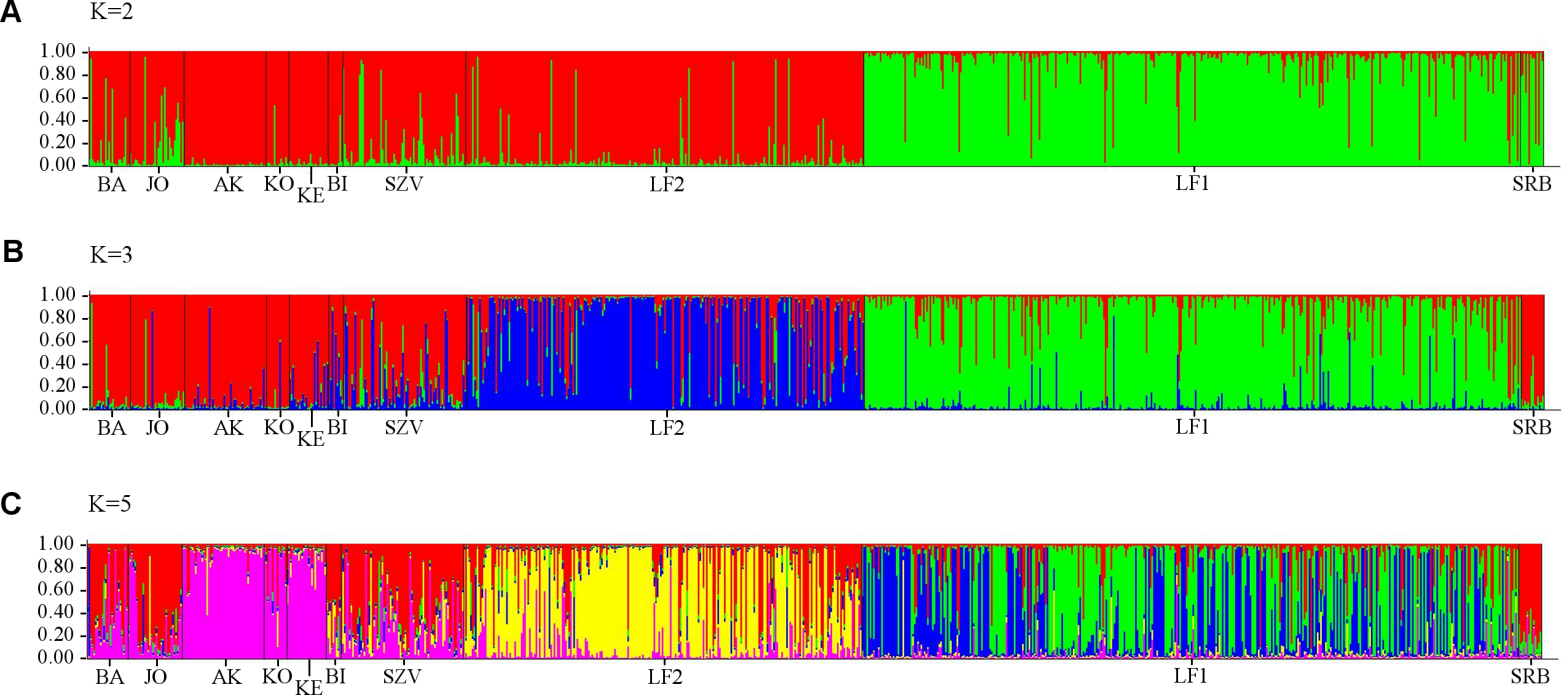
Bayesian individual assignment implemented using STRUCTURE for *K* = 2 clusters (A), *K* = 3 clusters (B) and *K* = 5 clusters (C) without using geographical area as a prior. The *y*-axis represents the probability of assignment of an individual to each cluster. BA, JO, KE, AK, KO and BI are Hungarian; SRB is a Serbian wild popuation; LF1-2 and SZV are hatcheries.

Analysis of all populations by STRUCTURE did not give a clear ΔK result, revealing two, three and five possible clusters of the sampled area. The highest probability of cluster numbers was at two, while the lowest at the five value (Fig. S1). Figure 4 shows a possible clustering that reflects the results of the NJ tree, but no clusters were restricted to one population. The individual assignment to the clusters made a distinction between the LF1 and LF2 broodstocks, and the assignment probability of the individuals in the SZV broodstock shows a transitional group between the other two broodstocks and the wild populations. The Serbian control also appears as a divergent group. Among the wild populations, AK and KE were the least variable groups.

**Figure 5:**
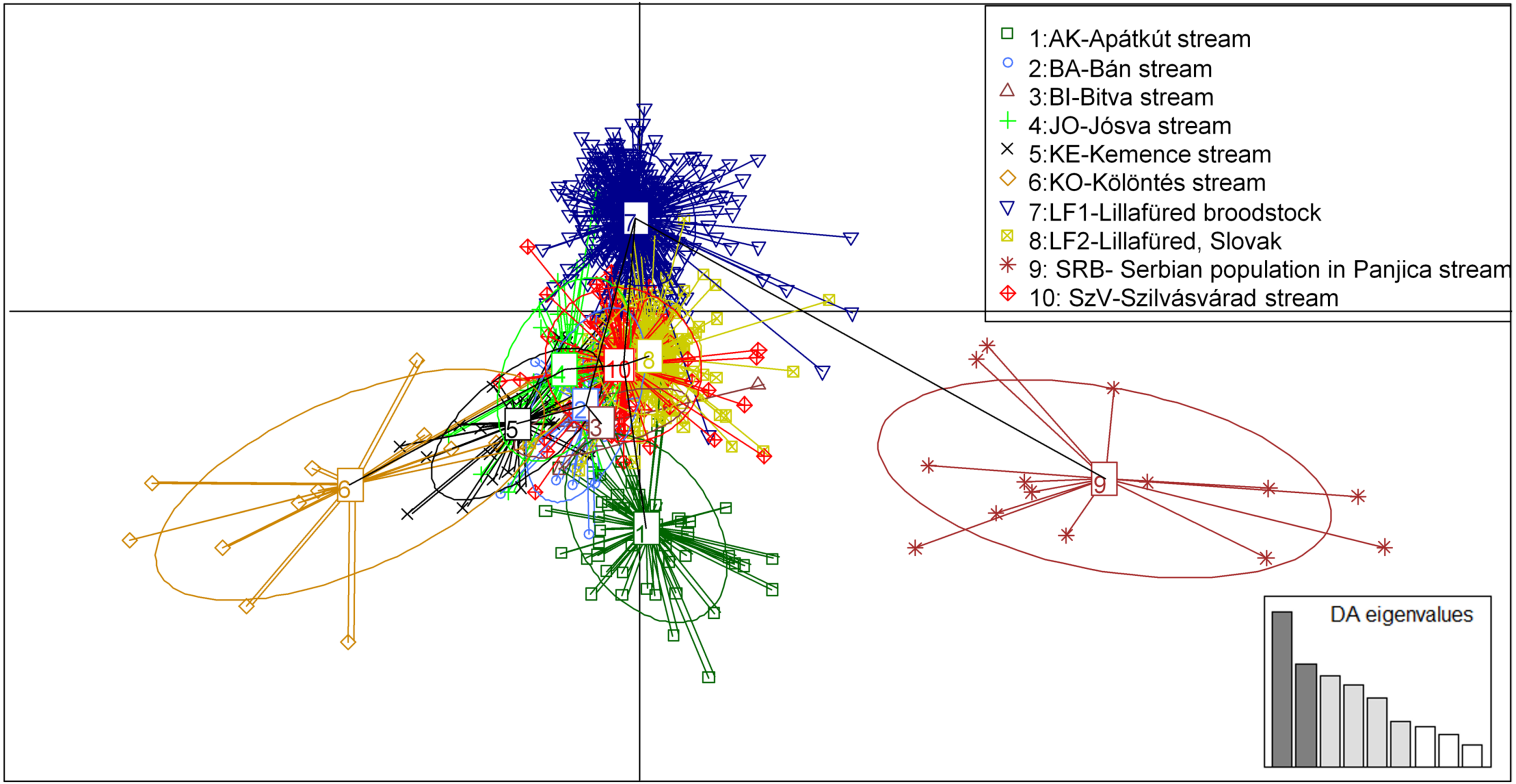
Principal component analysis (PCA) showing the sampled brown trout individuals: BA, JO, KE, AK, KO and BI are Hungarian; SRB is a Serbian wild populations; LF1-2 and SZV are hatchery populations. The *y*-axis represents 9.688 percentage, while the *X*-axis represents 6.744 percentage, of the total variance.

The PCA analysis showed very similar, but more structured clustering. The individuals were separated to three main groups by the horizontal axes while the distribution of the population were continuous along the vertical axes (Fig. 5). Both the STRUCTURE groupings and the principle component analysis showing moderate levels of separation among populations. However the populations, except for the Serbian one, have shown strong sub-structural sorting among the subgroups with overlapping individuals. PCA confirmed the results of STRUCTURE regrading of separated groups of SRB and LF1 populations. Individuals of the AK and KO differed from other wild populations which reflecting to the grouping of STURCTURE. The other populations were mainly distributed along the crossing of vertical axes.

AMOVA supports the results of STRUCTURE and PCA displaying a low variance among populations (10% of the whole variance, d.f. = 9) while 90% of the variance was found within populations (d.f. = 1,775). These in turn are mainly related to the variance within individuals (81% of whole variance, d.f. = 888) and only low variance was found among individuals (9%, d.f. = 878).

## Discussion

Very little information is available on the origin of the current brown trout populations in Hungary and what is known comes mostly from anecdotal information. The reason for this is that prior to World War I, these watercourses were mostly neglected by anglers and aquaculturists who preferred the more typical salmonid waters of the Carpathian mountains in today’s Slovakia, Ukraine and Romania. The interest in the salmonid waters of the low mountain ranges of today’s Hungary has increased following the Paris peace treaty of 1920. The Lillafüred trout farm has been founded in 1932 and the first shipment of trout eggs has arrived from Traismauer, Austria and Kláštor pod Znievom, Czechoslovakia (today Slovakia) in 1933. This broodstock was later the main supply of fish stocked into various streams in Hungary. Stocking of fish from this broodstock has been documented in the Bán and Jósva streams investigated in this study (Hoitsy, 2002).

This study is the first genetic diversity assessment of wild brown trout populations of Hungary. In addition, it offers further information regarding the two Hungarian broodstocks. The sample sizes in the case of some populations are low, especially in the KO, BI and SRB populations. These were however limited by the circumstances of sampling, hydrogeographical features and the abundance of fish in the given watercourses. For instance, in the Bittva stream (population BI) only three locations were available for sampling (100-m sections, each) at a distance of several km from each other. Effective analysis of genetic mixing using low-marker number has been proved in several studies, as well as in case of similar marker set as we used (Marić et al., 2006a; Horreo et al., 2015). However, different effect of gene drift on the applied markers should be considered (Henry & Ferguson, 1985; Hansen et al., 2000).

The mixed origin of the broodstocks and genetic admixture of Atlantic and Danubian lineages in the wild brown trout populations were revealed using multiple marker sets. High proportions of Atlantic haplotypes and alleles were found in the Danubian basin. All Atlantic haplotypes found in this study were frequently detected in other countries (i.e., At1 and At2 by Cortey et al., 2009; At3 by Duftner et al., 2003). These are widely distributed in the Atlantic basin (i.e., Spain, Norway, Iceland) as well as were found in hatcheries and mixed wild populations in the Danubian basin in Austria, Czech Republic, Slovakia and Italy (Duftner et al., 2003; Meraner et al., 2007; Cortey et al., 2009; Kohout et al., 2012; Fruciano et al., 2014; Gratton et al., 2014). Based on mitochondrial analyses the Danubian haplotypes were previously found in wild populations only in Austria and Slovakia (i.e.: Da1, Da2, Da3 by Duftner et al., 2003; Kohout et al., 2012). Two of our discovered haplotypes (Da4 and Da5) were found only in our hatcheries, and before this study both haplotypes appeared only in the Slovakian streams (Kohout et al., 2012). Haplotype Da6 has not been observed or reported previously, however it belongs to Danubian clade based on network analysis. Occurrence of this haplotype was restricted to a single individual found in the Kemence stream. Further sampling of this population should elucidate if this haplotype is indeed a private one to the KE stream.

Admixture of the different mitochondrial lineages has previously been detected in the Danubian drainage. An ancient colonization of the Danube river basin by an Atlantic lineage during or following the last glacial period was revealed by Schenekar, Lerceteau-Köhler & Weiss (2014) in Austrian streams, although the presence of these Atlantic haplotypes was only detected closer to the headwaters of Danube (Lerceteau-Köhler et al., 2013). Extensive distribution of the Atlantic lineage in the Danubian drainage system was also reported in the Czech Republic and Slovakia (Kohout et al., 2012), however, these are attributed to stocking activities. Given the limited information on the natural (non-anthropogenic) occurrence of the Atlantic lineage in the Danubian drainage system, its presence in Hungary can also be attributed to stocking.

Occasionally genetic admixture was documented by Hungarian hatcheries, as their brown trout breeders originated from Hungarian streams with indigenous brown trout stocks (supposedly belonging to the Danubian lineage) as well as from Austrian, German and Danish hatcheries that held breeders of Atlantic origin. Better performance of breeders with non-native alleles in hatchery conditions would explain their presence in all broodstocks (Horreo et al., 2015). Their fingerlings were stocked into Hungarian natural watercourses from 1933 until 2004, which contributed to a hybridization of the lineages. Furthermore the angling clubs in Hungary have stocked their territories with brown trout from various sources which have caused further gene flow between the lineages.

In addition the population genetic analyses of microsatellite data from field samples collected in different natural streams have revealed further information regarding the admixture. Only moderate differences were found between natural populations and the analysed broodstocks or among the various stream, (except for the Serbian population) based on *F*_ST_ values, as it was found in other studies, too (Vähä & Primmer, 2006). This can be a possible sign of admixture among the analysed groups. Admixture was confirmed by AMOVA analyses too, evidenced by a low level of differentiation observed among the populations, and high level of diversity were observed within the population. The inbreeding coefficients also indicated lower relatedness among the individuals than it would been expected under a model of random mating.

On the other hand, none of the analysed populations displayed significant deviation from the normal composition according to the results of Hardy–Weinberg equilibrium analyses. Only the PCR-RFLP marker loci in all broodstocks and a few streams showed a significant excess of heterozygosity, but in most cases the microsatellites also showed a tendency of heterozygosity excess. This could have been caused by anthropogenic effects (mixing of populations) or due to the low number of the available samples. These results refer to the post-stocking analyses of domesticated fishes in different rivers where fishes with poor performance were found (Weiss & Schmutz, 1999; Ferguson, 2007). Results of the sex-specific marker did not indicate any effect of admixture on the sex distributions in the broodstocks and one stream, whereas in the other streams female individuals were represented in less than 50 per cent of samples which can related to smaller sample sizes.

More detailed evidence of the admixture was shown by MJ tree, STRUCTURE plot and PCA results, which implies to anthropogenic transfer of fish among populations. However the STRUCTURE Harvester could not clearly determine the number of the genetic clusters, the colour plots of STRUCTURE showed the most possible differentiations of populations with five clusters. The genetically clustered groups are dispersed over the populations that correspond with the outcome of the Median-joining network analysis of mitochondrial haplotypes. It was confirmed by PCA analyses, that also showed five genetic groups (the SBR, AK, KO, LF1 were separated from the main cluster), but the classification of the individuals was more clear than in STRUCTURE. Because of these we recommend the usage of PCA analyses next to STRUCTURE. Although, we have used only a few microsatellites, the admixture among most streams and the cultured Szilvásvárad stock is evident. Only the geographically district Serbian population is separated (as it was expected), and the Apátkut, Kölöntés and the Lillafüred broodstock show lower overlaps with the genotype of the Szilvásvárad broodstock. However, all populations carry the elements of their ancient genetic backgrounds, which is shown by the relatively large number of private alleles (42), (private alleles were found in all populations, but the frequencies were low) and the fact that we could not identify non-admixed individuals. The identification of admixed and non-admixed (if any) individuals would be possible by increasing the number of analysed loci as it was illustrated by Hansen & Mensberg (2009). As a consequence of hybridization with non-native fish, reduced fitness of individuals in the wild has been reported (Muhlfeld et al., 2009).

Currently no regulations exist in Hungary on stocking of natural watercourses with non-indigenous populations beyond the level of species. Although stocking is considered an important conservation activity of autochthonous populations (Taylor, 1991), but the hybridization can cause reduced fitness as well as genetic variance of the native species (Allendorf et al., 2001). Thus, in an ideal case stocking should be carried out using only individuals originating from the local population by supportive breeding of these fishes. This strategy is widely spread in conservation of salmonids (Berrebi et al., 2000; Verspoor, Stradmeyer & Nielsen, 2007; Horreo et al., 2008). Due to the admixture of various lineages in Hungarian natural streams, this ideal case is not a viable option for the investigated streams, because the populations of the investigated streams are very small and there are no sufficient numbers of mature individuals to maintain the genetic diversity. Theoretically, if a unique population showing no signs of admixture was found in Hungary, we would suggest protection of that population and if stocking was necessary, this should be carried out only using that particular population. However, we cannot recommend this policy for the currently existing wild populations as they are in a Hardy-Weinberg equilibrium and do not represent unique genetic value.

## Conclusion

The first comprehensive study of Hungarian brown trout cultured broodstocks and wild streams revealed genetic admixture of the lineages in these populations, which could derive from natural or anthropogenic sources. Currently, there are no guidelines or common practices for stocking of small streams; however, the genetic background of these populations should be considered when developing conservation actions.

##  Supplemental Information

10.7717/peerj.5152/supp-1 Table S1 Estimates of evolutionary divergence between sequenced haplotypes The number of base substitutions per site from between sequences are shown, analyses were conducted using the Tamura 3-parameter model. Da, Danubian haplotypes, At, Atlantic haplotypes

10.7717/peerj.5152/supp-2 Table S2 Summary of the population genetic analyses of five microsatellite and two genomic PCR-RFLP loci for all sampled brown trout populations Pop, population, N, number of samples, Na, number of alleles, Nma, mean number of alleles, Neff, effective allele number, Ar, Allelic richness, He, expected heterozigosity, Ho observed heterozigosity, Sign, significant deviation, NA, not available, ns, not significant, ^∗^*P* < 005, ^∗∗^*P* < 0.01, ^∗∗∗^*P* < 0.001.

10.7717/peerj.5152/supp-3 Table S3 Private alleles of analysed microsatellite loci in two hatcheries and six wild populations in Hungary and in one wild Serbian population

10.7717/peerj.5152/supp-4 Table S4 Genetic data from all populations using various markers

10.7717/peerj.5152/supp-5 Figure S1 Graph of Δ*K* values of clustering analyses (rate of change in the log probability of data between successive *K* values), tested values: *K* = 1–12

10.7717/peerj.5152/supp-6 Supplemental Information 1 Sequences of Salmo trutta haplotypes originated form sampling area

## Additional Information and Declarations

### DNA Deposition

The following information was supplied regarding the deposition of DNA sequences: The sequences of nine haplotypes are accessible in Nucleotide database of National Center for Biotechnology Information (NCBI) with accession numbers from MG751088 to MG751096.

### Data Availability

The following information was supplied regarding data availability: The raw data are provided in Supplemental File.
