# Supplementary material for: The genetic status of the Hungarian brown trout populations: exploration of a blind spot on the European map of Salmo trutta studies
Source: PeerJ. 2018 Sep 21;6:e5152. doi: 10.7717/peerj.5152 (PMC6152457; doi:10.7717/peerj.5152)
Supplement: Table S1 — The number of base substitutions per site from between sequences are shown, analyses were conducted using the Tamura 3-parameter model. Da, Danubian haplotypes, At, Atlantic haplotypes [file peerj-06-5152-s001.docx]

**The genetic status of the Hungarian brown trout populations; exploration of a blind spot on the European map of *Salmo trutta* studies**

**Ágnes Ősz^1^, Ákos Horváth^1^, György Hoitsy^2^, Dóra Kánainé Sipos^1^, Szilvia Keszte^1^, Anna Júlia Sáfrány^1^, Saša Marić^3^, Csaba Palkó^4^, Balázs Tóth^5^, Béla Urbányi^1^, Balázs Kovács^1^**

**Supplemental Table 1**

**Table S1** Estimates of evolutionary divergence between sequenced haplotypes. The number of base substitutions per site from between sequences are shown, analyses were conducted using the Tamura 3-parameter model. Da: Danubian haplotypes, At: Atlantic haplotypes

|  | **Da1** | **Da2** | **Da3** | **Da4** | **Da5** | **Da6** | **At1** | **At2** |
| --- | --- | --- | --- | --- | --- | --- | --- | --- |
| **Da2** | 0.001 | - |  |  |  |  |  |  |
| **Da3** | 0.001 | 0.001 | - |  |  |  |  |  |
| **Da4** | 0.003 | 0.003 | 0.001 | - |  |  |  |  |
| **Da5** | 0.003 | 0.001 | 0.003 | 0.004 | - |  |  |  |
| **Da6** | 0.003 | 0.003 | 0.001 | 0.003 | 0.001 | - |  |  |
| **At1** | 0.009 | 0.011 | 0.011 | 0.012 | 0.012 | 0.012 | - |  |
| **At2** | 0.008 | 0.009 | 0.009 | 0.011 | 0.011 | 0.011 | 0.001 | - |
| **At3** | 0.011 | 0.012 | 0.012 | 0.013 | 0.013 | 0.013 | 0.001 | 0.003 |
