# Supplementary material for: The genetic status of the Hungarian brown trout populations: exploration of a blind spot on the European map of Salmo trutta studies
Source: PeerJ. 2018 Sep 21;6:e5152. doi: 10.7717/peerj.5152 (PMC6152457; doi:10.7717/peerj.5152)
Supplement: Table S2 — Pop, population, N, number of samples, Na, number of alleles, Nma, mean number of alleles, Neff, effective allele number, Ar, Allelic richness, He, expected heterozigosity, Ho observed heterozigosity, Sign, significant deviation, NA, not available, ns, not significant, ∗P < 005, ∗∗P < 0.01, ∗∗∗P < 0.001. [file peerj-06-5152-s002.docx]

**The genetic status of the Hungarian brown trout populations; exploration of a blind spot on the European map of *Salmo trutta* studies**

**Ágnes Ősz^1^, Ákos Horváth^1^, György Hoitsy^2^, Dóra Kánainé Sipos^1^, Szilvia Keszte^1^, Anna Júlia Sáfrány^1^, Saša Marić^3^, Csaba Palkó^4^, Balázs Tóth^5^, Béla Urbányi^1^, Balázs Kovács^1^**

**Supplemental Table 2**

**Table S2** Summary of the population genetic analyses of five microsatellite and two genomic PCR-RFLP loci for all sampled brown trout populations.

| **Pop** | **Loci** | **N** | **Na** | **Nma** | **Neff** | **Ar** |  | **He** | **Ho** | **Fis** | **Sign.** |
| --- | --- | --- | --- | --- | --- | --- | --- | --- | --- | --- | --- |
| **LF 1** | **MS Total** | **401** | **84** | **16.20** | **6.917** | **6.901** |  | **0.7721** | **0.7826** | **-0.0140** | **ns** |
|  | *BFRO002* |  | *5* |  | *1.859* | *2.940* |  | *0.4628* | *0.5138* | *-0.1100* | *ns* |
|  | *OMM1064* |  | *42* |  | *13.028* | *10.761* |  | *0.9244* | *0.9332* | *-0.0090* | *ns* |
|  | *Ssa408uos* |  | *17* |  | *10.141* | *9.185* |  | *0.9026* | *0.9497* | *-0.0520* | *ns* |
|  | *SsoSL417* |  | *11* |  | *6.148* | *6.798* |  | *0.8384* | *0.7688* | *0.0830* | **** |
|  | *SsoSL438* |  | *9* |  | *3.708* | *4.823* |  | *0.7322* | *0.7474* | *-0.0210* | *ns* |
|  | **PCR-RFLP Total** | **401** | **4** | **2** | **1.699** | **1.995** |  | **0.4057** | **0.4414** | **-0.0880** | ****** |
|  | *LDH-C1* |  | *2* |  | *1.874* | *2.000* |  | *0.4670* | *0.4763* | *-0.0200* | *ns* |
|  | *SL* |  | *2* |  | *1.524* | *1.989* |  | *0.3444* | *0.4065* | *-0.1800* | ***** |
| **LF 2** | **MS Total** | **243** | **93** | **18.60** | **7.004** | **6.848** |  | **0.7400** | **0.7805** | **-0.0550** | **ns** |
|  | *BFRO002* |  | *3* |  | *1.583* | *2.537* |  | *0.3691* | *0.4101* | *-0.1110* | *** |
|  | *OMM1064* |  | *43* |  | *16.030* | *11.958* |  | *0.9405* | *0.9337* | *0.0070* | *ns* |
|  | *Ssa408uos* |  | *29* |  | *9.186* | *9.395* |  | *0.8931* | *0.9558* | *-0.0700* | ***** |
|  | *SsoSL417* |  | *9* |  | *4.922* | *5.733* |  | *0.7987* | *0.8186* | *-0.0250* | *ns* |
|  | *SsoSL438* |  | *9* |  | *3.298* | *4.615* |  | *0.6984* | *0.7841* | *-0.1230* | *ns* |
|  | **PCR-RFLP Total** | **243** | **4** | **2** | **1.515** | **1.927** |  | **0.3140** | **0.3058** | **0.0260** | **ns** |
|  | *LDH-C1* |  | *2* |  | *1.218* | *1.853* |  | *0.1790* | *0.1818* | *-0.0160* | *ns* |
|  | *SL* |  | *2* |  | *1.812* | *2.000* |  | *0.4490* | *0.4298* | *0.0430* | *ns* |
| **SZV** | **MS Total** | **75** | **73** | **10.25** | **6.271** | **7.276** |  | **0.7621** | **0.7646** | **-0.0030** | **ns** |
|  | *BFRO002* |  | *3* |  | *1.615* | *2.539* |  | *0.3835* | *0.3973* | *-0.0360* | *ns* |
|  | *OMM1064* |  | *36* |  | *22.866* | *13.528* |  | *0.9627* | *0.9600* | *0.0030* | *ns* |
|  | *Ssa408uos* |  | *18* |  | *9.321* | *9.297* |  | *0.8987* | *0.9333* | *-0.0390* | *ns* |
|  | *SsoSL417* |  | *9* |  | *5.329* | *6.075* |  | *0.8178* | *0.7867* | *0.0380* | *ns* |
|  | *SsoSL438* |  | *7* |  | *3.868* | *4.940* |  | *0.7478* | *0.7458* | *0.0030* | *ns* |
|  | **PCR-RFLP Total** | **75** | **4** | **2** | **1.523** | **1.992** |  | **0.3454** | **0.3733** | **-0.0810** | **ns** |
|  | *LDH-C1* |  | *2* |  | *1.505* | *1.990* |  | *0.3379* | *0.4000* | *-0.1850* | *ns* |
|  | *SL* |  | *2* |  | *1.540* | *1.993* |  | *0.3529* | *0.3467* | *0.0180* | *ns* |
| **BA** | **MS Total** | **25** | **48** | **10.50** | **7.145** | **7.102** |  | **0.8121** | **0.8835** | **-0.0910** | **ns** |
|  | *BFRO002* |  | *4* |  | *2.456* | *3.339* |  | *0.6049* | *0.6800* | *-0.1270* | *ns* |
|  | *OMM1064* |  | *17* |  | *11.574* | *10.915* |  | *0.9322* | *0.9200* | *0.0130* | *ns* |
|  | *Ssa408uos* |  | *13* |  | *7.862* | *8.963* |  | *0.8906* | *0.9200* | *-0.0340* | *ns* |
|  | *SsoSL417* |  | *9* |  | *7.297* | *7.608* |  | *0.8821* | *0.9565* | *-0.0860* | *ns* |
|  | *SsoSL438* |  | *5* |  | *3.682* | *4.687* |  | *0.7504* | *0.9412* | *-0.2640* | *ns* |
|  | **PCR-RFLP Total** | **25** | **4** | **2** | **1.785** | **2.000** |  | **0.4493** | **0.5208** | **-0.1630** | **ns** |
|  | *LDH-C1* |  | *2* |  | *1.753* | *2.000* |  | *0.4388* | *0.5417* | *-0.2410* | *ns* |
|  | *SL* |  | *2* |  | *1.816* | *2.000* |  | *0.4598* | *0.5000* | *-0.0900* | *ns* |
| **JO** | **MS Total** | **33** | **56** | **12.25** | **7.446** | **6.866** |  | **0.6911** | **0.6798** | **0.0160** | **ns** |
|  | *BFRO002* |  | *3* |  | *1.167* | *2.096* |  | *0.1450* | *0.0909* | *0.3770* | *** |
|  | *OMM1064* |  | *23* |  | *16.133* | *12.424* |  | *0.9524* | *0.9394* | *0.0140* | *ns* |
|  | *Ssa408uos* |  | *15* |  | *8.037* | *8.966* |  | *0.8890* | *0.8485* | *0.0460* | *ns* |
|  | *SsoSL417* |  | *9* |  | *4.796* | *6.466* |  | *0.8041* | *0.7813* | *0.0290* | *ns* |
|  | *SsoSL438* |  | *6* |  | *2.859* | *4.377* |  | *0.6647* | *0.7391* | *-0.1150* | *ns* |
|  | **PCR-RFLP Total** | **33** | **4** | **2** | **1.316** | **1.944** |  | **0.2409** | **0.2779** | **-0.1570** | **ns** |
|  | *LDH-C1* |  | *2* |  | *1.234* | *1.905* |  | *0.1925* | *0.2121* | *-0.1030* | *ns* |
|  | *SL* |  | *2* |  | *1.398* | *1.982* |  | *0.2892* | *0.3438* | *-0.1920* | *ns* |
| **KE** | **MS Total** | **24** | **29** | **6.75** | **3.764** | **4.815** |  | **0.6852** | **0.6917** | **-0.0100** | **ns** |
|  | *BFRO002* |  | *2* |  | *1.800* | *2.000* |  | *0.4539* | *0.5000* | *-0.1040* | *ns* |
|  | *OMM1064* |  | *10* |  | *5.176* | *7.318* |  | *0.8256* | *0.7727* | *0.0650* | *ns* |
|  | *Ssa408uos* |  | *9* |  | *5.038* | *7.191* |  | *0.8193* | *0.8261* | *-0.0080* | *ns* |
|  | *SsoSL417* |  | *4* |  | *2.606* | *3.682* |  | *0.6294* | *0.5417* | *0.1420* | *ns* |
|  | *SsoSL438* |  | *4* |  | *3.143* | *3.886* |  | *0.6977* | *0.8182* | *-0.1780* | *ns* |
|  | **PCR-RFLP Total** | **24** | **4** | **2** | **1.825** | **2.000** |  | **0.4592** | **0.7083** | **-0.5610** | ******* |
|  | *LDH-C1* |  | *2* |  | *1.704* | *2.000* |  | *0.4220* | *0.5833* | *-0.3940* | *ns* |
|  | *SL* |  | *2* |  | *1.946* | *2.000* |  | *0.4965* | *0.8333* | *-0.7040* | ***** |
| **AK** | **MS Total** | **50** | **39** | **8.250** | **4.841** | **5.613** |  | **0.6757** | **0.6708** | **0.0070** | **ns** |
|  | *BFRO002* |  | *3* |  | *1.278* | *2.362* |  | *0.2198* | *0.2400* | *-0.0930* | *ns* |
|  | *OMM1064* |  | *14* |  | *8.982* | *8.973* |  | *0.8980* | *0.8958* | *0.0020* | *ns* |
|  | *Ssa408uos* |  | *9* |  | *3.783* | *6.343* |  | *0.7435* | *0.7447* | *-0.0020* | *ns* |
|  | *SsoSL417* |  | *8* |  | *5.782* | *6.229* |  | *0.8357* | *0.8333* | *0.0030* | *ns* |
|  | *SsoSL438* |  | *5* |  | *3.073* | *4.160* |  | *0.6814* | *0.6400* | *0.0610* | *ns* |
|  | **PCR-RFLP Total** | **50** | **4** | **2** | **1.432** | **1.983** |  | **0.3045** | **0.2900** | **0.0480** | **ns** |
|  | *LDH-C1* |  | *2* |  | *1.445* | *1.985* |  | *0.3109* | *0.2600* | *0.1650* | *ns* |
|  | *SL* |  | *2* |  | *1.419* | *1.981* |  | *0.2982* | *0.3200* | *-0.0740* | *ns* |
| **KO** | **MS Total** | **14** | **32** | **5.750** | **3.993** | **5.772** |  | **0.6292** | **0.6846** | **-0.0930** | **ns** |
|  | *BFRO002* |  | *3* |  | *1.446* | *2.844* |  | *0.3201* | *0.3571* | *-0.1210* | *ns* |
|  | *OMM1064* |  | *10* |  | *5.444* | *8.021* |  | *0.8466* | *0.9286* | *-0.1010* | *ns* |
|  | *Ssa408uos* |  | *8* |  | *6.500* | *7.471* |  | *0.8800* | *0.9231* | *-0.0510* | *ns* |
|  | *SsoSL417* |  | *8* |  | *6.480* | *8.000* |  | *0.8954* | *1.0000* | *-0.1250* | *ns* |
|  | *SsoSL438* |  | *3* |  | *1.244* | *2.524* |  | *0.2037* | *0.2143* | *-0.0540* | *ns* |
|  | **PCR-RFLP Total** | **14** | **4** | **2** | **1.517** | **1.882** |  | **0.2897** | **0.3214** | **-0.1140** | **ns** |
|  | *LDH-C1* |  | *2* |  | *1.960* | *2.000* |  | *0.5079* | *0.5714* | *-0.1300* | *ns* |
|  | *SL* |  | *2* |  | *1.074* | *1.643* |  | *0.0714* | *0.0714* | *0.0000* | *ns* |
| **BI** | **MS Total** | **9** | **25** | **4.750** | **3.415** | **5.000** |  | **0.7320** | **0.7111** | **0.0300** | **ns** |
|  | *BFRO002* |  | *2* |  | *1.976* | *2.000* |  | *0.5229* | *0.6667* | *-0.2970* | *ns* |
|  | *OMM1064* |  | *6* |  | *4.500* | *6.000* |  | *0.8235* | *0.8889* | *-0.0850* | *ns* |
|  | *Ssa408uos* |  | *7* |  | *4.378* | *7.000* |  | *0.8170* | *0.6667* | *0.1930* | *ns* |
|  | *SsoSL417* |  | *5* |  | *3.767* | *5.000* |  | *0.7778* | *0.6667* | *0.1500* | *ns* |
|  | *SsoSL438* |  | *5* |  | *3.115* | *5.000* |  | *0.7190* | *0.6667* | *0.0770* | *ns* |
|  | **PCR-RFLP Total** | **9** | **3** | **1.5** | **1.264** | **1.500** |  | **0.1830** | **0.2222** | **-0.2310** | **ns** |
|  | *LDH-C1* |  | *2* |  | *1.528* | *2.000* |  | *0.3660* | *0.4444* | *-0.2310* | *ns* |
|  | *SL* |  | *1* |  | *1.000* | *1.000* |  | *0.0000* | *0.0000* | *NA* | *NA* |
| **SRB** | **MS Total** | **14** | **29** | **3.750** | **2.118** | **4.805** |  | **0.5651** | **0.5714** | **-0.0120** | **ns** |
|  | *BFRO002* |  | *2* |  | *1.074* | *1.643* |  | *0.0714* | *0.0714* | *0.0000* | *ns* |
|  | *OMM1064* |  | *11* |  | *4.900* | *8.572* |  | *0.8254* | *0.6429* | *0.2280* | *ns* |
|  | *Ssa408uos* |  | *8* |  | *3.409* | *6.333* |  | *0.7328* | *0.7857* | *-0.0750* | *ns* |
|  | *SsoSL417* |  | *6* |  | *3.920* | *5.477* |  | *0.7725* | *0.9286* | *-0.2110* | *ns* |
|  | *SsoSL438* |  | *2* |  | *1.690* | *2.000* |  | *0.4233* | *0.4286* | *-0.0130* | *ns* |
|  | **PCR-RFLP Total** | **14** | **2** | **1** | **1.000** | **1.000** |  | **0.0000** | **0.0000** | **NA** | **NA** |
|  | *LDH-C1* |  | *1* |  | *1.000* | *1.000* |  | *0.0000* | *0.0000* | *NA* | *NA* |
|  | *SL* |  | *1* |  | *1.000* | *1.000* |  | *0.0000* | *0.0000* | *NA* | *NA* |

Pop: population, N: number of samples, Na: number of alleles, Nma: mean number of alleles, Neff: effective allele number, Ar: Allelic richness, He:expected heterozigosity, Ho observed heterozigosity, Sign: significant deviation, NA=not available, ns=not significant, * P<005, ** P<0.01, *** P<0.001
