# Supplementary material for: The genetic status of the Hungarian brown trout populations: exploration of a blind spot on the European map of Salmo trutta studies
Source: PeerJ. 2018 Sep 21;6:e5152. doi: 10.7717/peerj.5152 (PMC6152457; doi:10.7717/peerj.5152)
Supplement: Table S3 [file peerj-06-5152-s003.docx]

**The genetic status of the Hungarian brown trout populations; Exploration of a blind spot on the European map of *Salmo trutta* studies**

**Ágnes Ősz^1^, Ákos Horváth^1^, György Hoitsy^2^, Dóra Kánainé Sipos^1^, Szilvia Keszte^1^, Anna Júlia Sáfrány^1^, Saša Marić^3^, Csaba Palkó^4^, Balázs Tóth^5^, Béla Urbányi^1^, Balázs Kovács^1^**

**Supplemental Table 3**

**Table S3** Private alleles of analysed microsatellite loci in two hatcheries and six wild populations in Hungary and in one wild Serbian population.

| **Population** | **Locus** | **Allele** | **Frequency** |
| --- | --- | --- | --- |
| **LF1** | BFRO002 | 110 | 0.004 |
|  | OMM1064 | 158 | 0.001 |
|  |  | 164 | 0.006 |
|  |  | 216 | 0.003 |
|  |  | 234 | 0.001 |
|  |  | 239 | 0.001 |
|  |  | 263 | 0.001 |
|  |  | 346 | 0.003 |
|  | SsoSL417 | 169 | 0.001 |
|  | SsoSL438 | 93 | 0.003 |
|  |  | 97 | 0.162 |
| **LF2** | OMM1064 | 186 | 0.123 |
|  |  | 225 | 0.009 |
|  |  | 286 | 0.003 |
|  |  | 296 | 0.003 |
|  |  | 337 | 0.003 |
|  | Ssa408uos | 213 | 0.002 |
|  |  | 225 | 0.007 |
|  |  | 269 | 0.002 |
|  |  | 281 | 0.002 |
|  |  | 285 | 0.002 |
|  |  | 303 | 0.002 |
| **SZV** | OMM1064 | 331 | 0.007 |
| **BI** | OMM1064 | 202 | 0.167 |
|  | Ssa408uos | 245 | 0.056 |
|  | SsoSL417 | 196 | 0.056 |
| **JO** | OMM1064 | 250 | 0.061 |
|  |  | 272 | 0.015 |
|  |  | 277 | 0.030 |
|  |  | 316 | 0.106 |
|  | Ssa408uos | 253 | 0.045 |
|  |  | 260 | 0.015 |
|  |  | 273 | 0.015 |
| **KE** | OMM1064 | 162 | 0.045 |
| **AK** | OMM1064 | 166 | 0.104 |
|  |  | 224 | 0.052 |
| **KO** | OMM1064 | 174 | 0.321 |
|  | Ssa408uos | 289 | 0.231 |
| **BA** | OMM1064 | 252 | 0.020 |
|  | Ssa408uos | 284 | 0.020 |
| **SRB** | OMM1064 | 206 | 0.036 |
|  | Ssa408uos | 309 | 0.250 |
