# Supplementary figures and images for: The genetic status of the Hungarian brown trout populations: exploration of a blind spot on the European map of Salmo trutta studies
Source: PeerJ. 2018 Sep 21;6:e5152. doi: 10.7717/peerj.5152 (PMC6152457; doi:10.7717/peerj.5152)

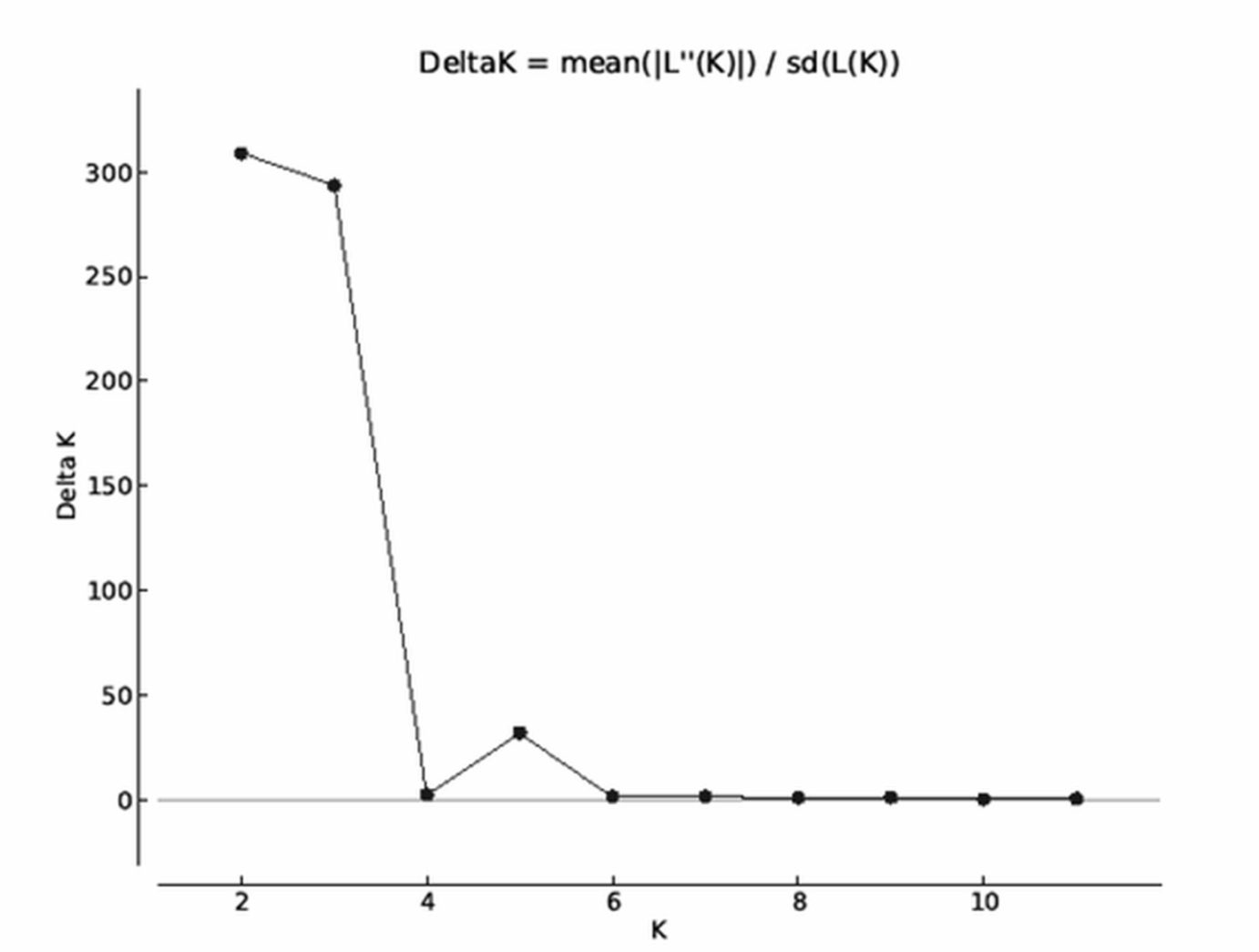

Supplement: Figure S1 [file peerj-06-5152-s005.png]
